# Longitudinal Surveillance of Gastric Polyposis in Familial Adenomatous Polyposis: Incidence, Progression, and Endoscopic Outcomes

**DOI:** 10.1002/ueg2.70216

**Published:** 2026-04-13

**Authors:** Robert Hüneburg, Julia Gieffers‐Löwen, Stefan Aretz, Katrin van Beekum, Sonja Haas, Anne‐Sophie Layritz, Tim Marwitz, Dominik Heling, Glen Kristiansen, Christian P. Strassburg, Jacob Nattermann

**Affiliations:** ^1^ Department of Internal Medicine I University Hospital Bonn Bonn Germany; ^2^ National Center for Hereditary Tumor Syndromes University Hospital Bonn Bonn Germany; ^3^ Institute of Human Genetics Medical Faculty University of Bonn Bonn Germany; ^4^ Institute of Pathology University Hospital Bonn Bonn Germany

**Keywords:** endoscopic management, familial adenomatous polyposis, fundic gland polyps, gastric cancer, gastric polyposis, upper gastrointestinal surveillance

## Abstract

**Background:**

Gastric manifestations of familial adenomatous polyposis (FAP) have traditionally been considered of limited relevance in Western populations, with surveillance focused on duodenal disease. Recent data suggest that progressive gastric polyposis, particularly in the proximal stomach, may be associated with dysplasia and carcinoma. We aimed to characterize longitudinal gastric phenotype evolution and compartment‐specific risk in a contemporary Western FAP cohort.

**Methods:**

We conducted a retrospective longitudinal cohort study of patients with genetically confirmed FAP undergoing structured upper gastrointestinal surveillance between 2019 and 2023. Gastric phenotypes were assessed separately for the fundus/corpus and antrum. Fundic gland polyp (FGP) burden was recorded semiquantitatively. Gastric dysplasia and carcinoma were analyzed as compartment‐specific outcomes.

**Results:**

A total of 299 patients (1281 upper endoscopies; mean follow‐up 2.6 years) were included. Fundic/corpus polyposis was present in 81.9% of patients at the baseline. During follow‐up, the FGP burden increased substantially, with more than 100 polyps observed in 52.2% of patients during their 5th‐year follow‐up. Fundic/corpus dysplasia increased from 10.7% at baseline to 40.0% (annual point prevalence) by year 5 and was independently associated with a baseline FGP burden > 200 polyps. Gastric adenocarcinoma occurred in 6 patients (2.0%), predominantly in the proximal stomach and in the setting of extensive polyposis, with poor outcomes. In contrast, antral dysplasia followed a more indolent course.

**Discussion:**

In FAP, gastric disease is dynamic and compartment‐specific. Progressive fundic gland polyposis is associated with a high‐risk phenotype for proximal gastric neoplasia, supporting phenotype‐driven gastric surveillance strategies.

## Key Summary

- Summarize the established knowledge on this subject
  ◦ In familial adenomatous polyposis (FAP), upper gastrointestinal surveillance has traditionally focused on duodenal neoplasia, while gastric manifestations had limited relevance in Western populations.
  ◦ Fundic gland polyps (FGPs) are common in FAP and frequently harbor low‐grade dysplasia, but their clinical significance has long been debated.
  ◦ Recent Western series report proximal gastric adenocarcinomas arising in the setting of extensive fundic gland polyposis, often diagnosed at an advanced stage despite surveillance.
  ◦ Longitudinal data on gastric phenotype evolution and compartment‐specific neoplastic risk in FAP remain scarce.
- What are the significant and/or new findings of this study?
  ◦ In a large contemporary Western FAP cohort (*n* = 299; 1281 endoscopies), fundic gland polyposis was highly prevalent (81.9%) and progressed substantially over time.
  ◦ Increasing the fundic gland polyp burden, particularly more than 200 polyps at the baseline, was independently associated with fundus/corpus dysplasia.
  ◦ Gastric adenocarcinoma occurred in 2.0% of patients, predominantly in the proximal stomach and in the setting of extensive polyposis, with poor outcomes.
  ◦ Antral dysplasia followed a distinct and more indolent course, supporting compartment‐specific, phenotype‐driven gastric surveillance strategies.

## 1 Introduction

Familial adenomatous polyposis (FAP) is a hereditary cancer predisposition syndrome caused by pathogenic germline variants in the APC gene and is characterized by the development of numerous colorectal adenomas and an almost inevitable risk of colorectal cancer without preventive intervention [1]. In addition to colorectal disease, patients with FAP are at an increased risk of extracolonic manifestations, including neoplasia of the upper gastrointestinal tract [2].

Historically, upper gastrointestinal surveillance and risk stratification in FAP have focused predominantly on duodenal and ampullary neoplasia, which were long considered the principal drivers of upper gastrointestinal cancer‐related morbidity and mortality [2, 3]. Duodenal adenomas occur in up to 90% of patients with FAP, and duodenal cancer risk has therefore shaped surveillance strategies and guideline recommendations for decades [3, 4].

While duodenal neoplasia represents the leading cause of upper gastrointestinal cancer‐related morbidity in FAP, gastric manifestations were traditionally regarded as clinically less relevant in Western populations, largely because early series reported few gastric cancers despite a high prevalence of gastric polyps [2]. Gastric involvement in FAP encompasses a heterogeneous spectrum of glandular lesions, including fundic gland polyps (FGPs), gastric adenomas of various histologic subtypes, and less commonly hyperplastic or pyloric gland lesions [5].

FGPs are the most common gastric manifestation of FAP and are identified in the majority of patients in contemporary Western cohorts [6, 7].

Duodenal and ampullary neoplasia have long dominated upper gastrointestinal risk stratification in FAP, with duodenal adenomas occurring in up to 90% of patients and shaping guideline recommendations for decades [2, 3, 4]. Gastric manifestations were traditionally regarded as clinically less relevant in Western populations. Gastric involvement in FAP encompasses a heterogeneous spectrum of glandular lesions, including fundic gland polyps (FGPs), gastric adenomas of various histologic subtypes, and less commonly hyperplastic or pyloric gland lesions [5]. FGPs are the most common gastric manifestation and are identified in the majority of patients in contemporary Western cohorts [6, 7, 8].

Although FGPs were long considered largely benign, multiple studies have demonstrated that low‐grade dysplasia is present in approximately 30%–60% of FGPs in FAP, while high‐grade dysplasia is uncommon but consistently reported, occurring in up to 1%–10% of cases [5, 9]. Despite these histological findings, the clinical relevance of FGP‐associated dysplasia was historically considered limited, and gastric surveillance strategies therefore remained secondary to duodenal assessment and largely lesion‐based, with little emphasis on gastric phenotype evolution, lesion burden, or compartment‐specific cancer risk [4].

More recent data from Western expert centers have challenged this view. Contemporary case series and cohort studies have reported gastric adenocarcinomas in patients with FAP arising predominantly in the fundus and corpus, frequently in the setting of extensive fundic gland polyposis characterized by carpeting or polypoid mounds [9, 10, 11, 12]. These cancers are often diagnosed at an advanced stage despite regular endoscopic surveillance and are associated with poor outcomes [9, 12].

Several morphologic features have been proposed as predictors of neoplastic progression in the proximal stomach, including large polyp size, extensive or diffuse polyp distribution, high‐grade dysplasia, and the presence of gastric adenomas [11]. However, most available studies are cross‐sectional or limited to small cohorts, and longitudinal data capturing the natural history and progression of FGP burden over time remain scarce.

In contrast to the proximal stomach, antral lesions in FAP appear to follow a different biological trajectory. Gastric adenomas arising in the antrum are less frequent, often detected incidentally, and generally demonstrate a more indolent course [13]. Malignant transformation in the antrum is rare, and when present, lesions are more often amenable to curative endoscopic resection.

Current guidelines recommend upper GI surveillance in FAP but provide limited granularity regarding gastric risk stratification beyond lesion size and histology, reflecting the paucity of longitudinal data [4, 14, 15]. Surveillance intervals and management strategies are therefore largely extrapolated from duodenal disease or based on expert opinion, and do not consistently account for gastric phenotype evolution or compartment‐specific risk. As a result, clinically relevant progression in the proximal stomach may remain underappreciated, while other lesions may be overtreated.

In this context, we aimed to comprehensively characterize the prevalence, longitudinal evolution, and clinical relevance of gastric glandular polyps and associated neoplasia in a large, contemporary cohort of patients with familial adenomatous polyposis undergoing modern endoscopic surveillance. Our objectives were to (i) delineate the temporal progression of fundic and corpus FGP burden, (ii) quantify the incidence and compartment‐specific evolution of gastric dysplasia and carcinoma, (iii) identify clinical and endoscopic factors associated with neoplastic transformation, and (iv) evaluate endoscopic management strategies and procedural outcomes. By integrating longitudinal phenotypic trajectories with patient‐level risk analyses, this study seeks to provide an evidence base for phenotype‐adapted, compartment‐specific gastric surveillance in FAP.

## 2 Methods

### 2.1 Study Design and Patient Population

We conducted a retrospective cohort study of genetically confirmed FAP (pathogenic/likely pathogenic APC variant and ACMG Class IV/V) patients undergoing structured upper GI surveillance at our tertiary referral center between 2019 and 2023, reflecting contemporary practice after recognition of increased gastric cancer risk in Western FAP populations. Patients with prior gastrectomy were excluded; longitudinal analyses included patients with ≥ 2 endoscopies. The study was approved by the local ethics committee (415/19), and all patients provided written consent.

### 2.2 Endoscopic Surveillance Protocol

Upper gastrointestinal surveillance was performed using high‐definition white‐light endoscopy. Virtual chromoendoscopy techniques, specifically linked‐color imaging (LCI) and blue‐light imaging (BLI), were used as adjunctive imaging modalities at the discretion of the endoscopist, particularly for lesion detection and characterization.

Examinations were performed by experienced FAP endoscopists using a systematic compartment‐based inspection with structured documentation of lesion number, size, morphology, and distribution. Surveillance intervals were individualized based on phenotypic findings, with shorter intervals applied in patients with extensive polyposis and/or histologically confirmed dysplasia.

Lesions were selected for resection based on two complementary strategies: (i) targeted resection of all macroscopically suspicious areas identified by focal discoloration, surface irregularity, or abnormal vascular or surface pattern on white‐light endoscopy and/or virtual chromoendoscopy; and (ii) systematic field sampling of endoscopically unremarkable areas within the polyposis field in patients with extensive polyposis or carpeting, to account for the risk of dysplasia within macroscopically inconspicuous regions. In addition, larger polyps were preferentially removed to obtain representative histological assessment. This combined strategy of targeted resection and representative field sampling is consistent with previously described endoscopic surveillance approaches in patients with familial adenomatous polyposis [16]. Tissue acquisition was performed exclusively by snare‐based resection rather than forceps biopsy throughout, allowing histopathological evaluation of complete polyp specimens. Clearly demarcated lesions suspicious for dysplasia were resected with en‐bloc intent using endoscopic mucosal resection (EMR) or, where appropriate, endoscopic submucosal dissection (ESD), whereas diffuse carpet‐like polyposis without a defined focal lesion was managed by cold snare polypectomy or limited piecemeal EMR for representative sampling and field reduction.

### 2.3 Assessment of Fundic Gland Polyp Burden

Fundic and the corpus gland polyp burden was assessed endoscopically and recorded as an estimated total count per procedure, based on a systematic inspection of the fundus and corpus. Polyp burden was documented as part of routine clinical reporting by experienced endoscopists with expertise in FAP surveillance.

For analytical purposes, the polyp burden was categorized into predefined ordinal groups (0, 1–50, 51–100, 101–200, 201–400, and more than 400 polyps), reflecting standardized institutional documentation practice. For selected analyses, extensive fundic gland polyposis was defined using a dichotomous threshold of more than 200 polyps per examination.

### 2.4 Histopathological Evaluation

All biopsy and resection specimens were evaluated by experienced gastrointestinal pathologists with expertise in hereditary gastrointestinal tumor syndromes. Dysplasia was classified as low‐grade dysplasia (LGD) or high‐grade dysplasia (HGD) according to the Vienna classification for gastrointestinal epithelial neoplasia. For the purpose of longitudinal risk assessment, gastric dysplasia was analyzed as a composite end point, irrespective of specific histological subcategorization, reflecting routine clinical reporting and the study's focus on phenotypic progression rather than lesion subtype. Gastric adenocarcinomas were staged according to the current TNM classification. For all analyses, dysplasia outcomes were evaluated separately for the fundus/corpus and the antrum to account for compartment‐specific differences in the lesion phenotype and malignant potential.

### 2.5 Endoscopic Resection Techniques and Adverse Events

Endoscopic therapy was performed for selected gastric lesions at the discretion of the treating endoscopist. Resection techniques included cold snare polypectomy, endoscopic mucosal resection (EMR), and in selected cases, endoscopic submucosal dissection (ESD). For each procedure, the resection technique and whether resection was performed en bloc or piecemeal were documented.

Procedure‐related adverse events were prospectively recorded in the institutional endoscopy reporting system and classified according to established AGREE consensus definitions for adverse events in gastrointestinal endoscopy [17]. Bleeding events were classified as minor or major. Minor bleeding was defined as intraprocedural or delayed bleeding controlled endoscopically without clinical sequelae. Major bleeding was defined as bleeding requiring blood transfusion, intensive care unit monitoring, or repeat endoscopic intervention. Perforation was defined as a full‐thickness defect identified endoscopically and/or confirmed radiologically.

### 2.6 Statistical Analyses

Annual dysplasia prevalence was estimated using a ± 6‐month window around each integer year of follow‐up to accommodate variable surveillance intervals across patients. Cumulative incidence was estimated using the Kaplan‐Meier method. Multivariable logistic regression analyses evaluated patient‐level ever/never dysplasia outcomes, adjusted for age, sex, PPI use, and *H. pylori* status. Sensitivity analyses using alternative FGP burden thresholds (> 100, > 200, > 400 polyps) and an ordinal burden category model are presented in Supporting Information S1: Table S8. Sensitivity analyses adjusting for the total number of surveillance endoscopies and the mean surveillance interval per patient are presented in Supporting Information S1: Table S9. All analyses were performed using R version 4.3.2.

## 3 Results

### 3.1 Cohort Characteristics and Surveillance

A total of 299 patients with familial adenomatous polyposis (FAP) from 214 distinct families underwent upper gastrointestinal surveillance at our tertiary referral center between January 2019 and December 2023. The cohort comprised 176 women (58.9%) and 123 men (41.1%), with a mean age of 39 ± 14 years (range 14–73 years) at the first upper endoscopy.

Overall, 1281 upper gastrointestinal endoscopies were performed, corresponding to a mean of 4.3 procedures per patient (range 1–16). The mean observational period was 2.6 years (range 0–5 years). Longitudinal analyses of phenotypic progression were restricted to the 267 patients who underwent at least two surveillance endoscopies.

The reduction in patient numbers across annual time points reflects administrative censoring due to the fixed study period (2019–2023) rather than selective clinical dropout; patients enrolled later in the observation window had fewer years of available follow‐up by the time of data cut‐off. A baseline comparison of patients with complete 5‐year follow‐up versus shorter available follow‐up is provided in Supporting Information S1: Table S10.

### 3.2 Fundic/Corpus Phenotype: Baseline Status and Longitudinal Progression

At baseline (Year 0), FGPs were the predominant gastric phenotype and were identified in 245 of 299 patients (81.9%) (Table 1). Most patients exhibited a low‐to‐moderate FGP burden, with 38.5% presenting with 1–50 polyps and 15.4% with 51–100 polyps. A fundic gland polyp burden exceeding 100 polyps was observed in 28.1% of patients, whereas profound polyposis (> 400 polyps) was uncommon (2.7%).

**TABLE 1:** Annual fundic gland polyp burden during longitudinal surveillance in patients with familial adenomatous polyposis.

Year	n	No FGP	1–50 FGP	51–100 FGP	101–200 FGP	201–400 FGP	> 400 FGP	Any FGP
Year 0	299	54 (18.1%)	115 (38.5%)	46 (15.4%)	58 (19.4%)	18 (6.0%)	8 (2.7%)	245 (81.9%)
Year 1	179	20 (11.2%)	68 (38.0%)	34 (19.0%)	37 (20.7%)	12 (6.7%)	8 (4.5%)	159 (88.8%)
Year 2	198	31 (15.7%)	66 (33.3%)	43 (21.7%)	39 (19.7%)	12 (6.1%)	7 (3.5%)	167 (84.3%)
Year 3	153	19 (12.4%)	45 (29.4%)	27 (17.6%)	43 (28.1%)	9 (5.9%)	10 (6.5%)	134 (87.6%)
Year 4	110	7 (6.4%)	37 (33.6%)	17 (15.5%)	37 (33.6%)	5 (4.5%)	7 (6.4%)	103 (93.6%)
Year 5	90	10 (11.1%)	26 (28.9%)	13 (14.4%)	31 (34.4%)	5 (5.6%)	5 (5.6%)	80 (88.9%)

*Note: n* per year = patients undergoing endoscopy in ± 6‐month window.

Neoplastic changes were already detectable at baseline: low‐grade dysplasia (LGD) of the fundus/corpus was identified in 10.7% of patients. Neither high‐grade dysplasia (HGD) nor gastric adenocarcinoma was observed at the initial examination (Table 2).

**TABLE 2:** Incidence of fundus/corpus gastric dysplasia during longitudinal surveillance.

Year	*n*	New LGD	Cum. LGD	New HGD	Cum. HGD	New any	Cum. Any
Year 0	299	32 (10.7%)	32 (10.7%)	0 (0.0%)	0 (0.0%)	32 (10.7%)	32 (10.7%)
Year 1	179	14 (4.7%)	46 (15.4%)	1 (0.3%)	1 (0.3%)	15 (5.0%)	47 (15.7%)
Year 2	198	17 (5.7%)	63 (21.1%)	2 (0.7%)	3 (1.0%)	19 (6.4%)	66 (22.1%)
Year 3	153	17 (5.7%)	80 (26.8%)	1 (0.3%)	4 (1.3%)	18 (6.0%)	84 (28.1%)
Year 4	110	9 (3.0%)	89 (29.8%)	3 (1.0%)	7 (2.3%)	12 (4.0%)	96 (32.1%)
Year 5	90	17 (5.7%)	106 (35.5%)	2 (0.7%)	9 (3.0%)	19 (6.4%)	115 (38.5%)

*Note:* Cells show *n* (% of patients of the overall cohort, *n* = 299). ‘New’ indicates first occurrence since the previous surveillance interval. ‘Any dysplasia’ includes low‐grade or high‐grade dysplasia detected in the fundus or corpus.

Abbreviations: HGD = high‐grade dysplasia; LGD = low‐grade dysplasia.

During longitudinal follow‐up, a pronounced shift toward higher FGP burdens was observed (Table 1). Among patients with surveillance extending to Year 5, the prevalence of a fundic gland polyp burden exceeding 100 polyps increased to 52.2%, including 30.0% with 101–200 FGPs, 11.1% with 201–400 FGPs, and 11.1% with more than 400 FGPs.

This morphological progression was accompanied by an accumulation of neoplastic changes. The annual point prevalence of fundic/corpus dysplasia increased steadily from 10.7% at baseline to 40.0% in Year 5 (Supporting Information S1: Table S1). LGD remained the predominant finding, while HGD emerged during surveillance, reaching a prevalence of 2.2% by Year 5. Cumulative incidence analyses demonstrated a steady accrual of fundic/corpus dysplasia over time, both in the overall cohort (Table 2) and among patients who have completed their 5th‐year follow‐up (Supporting Information S1: Table S2). Detailed longitudinal analyses stratified by surveillance duration and gastric compartment are provided in the Supporting Information S1: Tables.

### 3.3 Antral Phenotype: Baseline Status and Longitudinal Progression

At baseline, antral adenomas were detected in 27 of 299 patients (9.0%), all classified as LGD (Table 3). During follow‐up, cumulative incidence of antral dysplasia increased steadily, reaching 23.1% by Year 5 in the overall cohort (Table 3). Comparable estimates were observed in patients after their 5‐year follow‐up. The majority of lesions remained low‐grade, whereas HGD was identified in a small subset of patients (1.1%). Annual point prevalence and cumulative incidence of antral dysplasia stratified by complete 5‐year follow‐up are shown in Supporting Information S1: Tables S3 and S4.

**TABLE 3:** Cumulative incidence of antral dysplasia during longitudinal surveillance.

Year	*n*	New LGD	Cum. LGD	New HGD	Cum. HGD	New any	Cum. Any
Year 0	299	27 (9.0%)	27 (9.0%)	0 (0.0%)	0 (0.0%)	27 (9.0%)	27 (9.0%)
Year 1	179	13 (4.3%)	40 (13.4%)	1 (0.3%)	1 (0.3%)	14 (4.7%)	41 (13.7%)
Year 2	198	10 (3.3%)	50 (16.7%)	0 (0.0%)	1 (0.3%)	10 (3.3%)	51 (17.1%)
Year 3	153	6 (2.0%)	56 (18.7%)	2 (0.7%)	3 (1.0%)	7 (2.3%)	58 (19.4%)
Year 4	110	6 (2.0%)	62 (20.7%)	0 (0.0%)	3 (1.0%)	6 (2.0%)	64 (21.4%)
Year 5	90	5 (1.7%)	67 (22.4%)	0 (0.0%)	3 (1.0%)	5 (1.7%)	69 (23.1%)

*Note:* Cells show *n* (% of patients of the overall cohort, *n* = 299). Cumulative incidence reflects first detection of dysplasia during follow‐up. ‘Any dysplasia’ includes low‐grade or high‐grade dysplasia detected in the antrum.

Abbreviations: HGD = high‐grade dysplasia; LGD = low‐grade dysplasia.

### 3.4 Any Gastric Dysplasia

When fundic/corpus and antral compartments were considered jointly, cumulative incidence analyses demonstrated that nearly half of all patients developed gastric dysplasia during follow‐up (Supporting Information S1: Table S7), with higher cumulative incidence among patients with complete 5‐year surveillance (Supporting Information S1: Table S6). Annual prevalence estimates are provided in Supporting Information S1: Table S5.

### 3.5 Gastric Carcinoma

**FIGURE 1:**
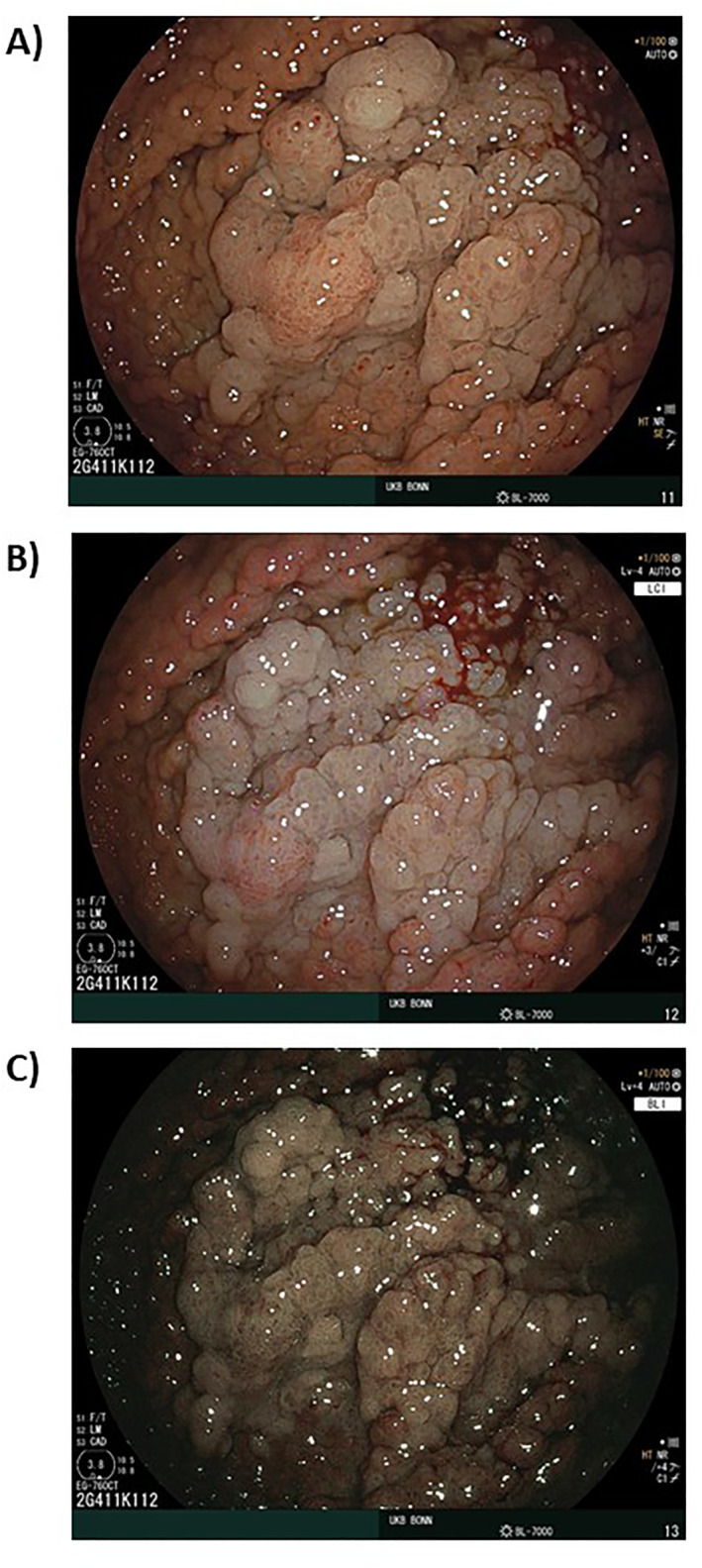
Gastric carcinoma arising within an area of carpeting fundic gland polyposis in a patient with familial adenomatous polyposis. The same lesion is shown in (A) white‐light imaging, (B) linked color imaging (LCI), which accentuates color contrast and delineates a focal erythematous area within the densely polyposis mucosa, and (C) blue light imaging (BLI), which highlights an irregular microsurface and a microvascular pattern suspicious for invasive neoplasia.

Gastric adenocarcinoma was diagnosed in 6 patients (2.0%) during the study period (Table 4 and Figure 1). Five tumors originated in the fundus or corpus, whereas one carcinoma was located in the antrum.

**TABLE 4:** Clinical characteristics, management, and outcomes of gastric adenocarcinoma in patients with familial adenomatous polyposis under surveillance (*n* = 6).

Age at diagnosis (years)	Sex	APC pathogenic variant	Previous colorectal surgery	No. Previous upper endoscopies	Interval since last endoscopy	Findings at previous endoscopy	Spigelman stage (points)	Tumor location	Lesion size (mm)	Index histology (LGD/HGD/Ca)	Definitive treatment	Final pathology	Metastatic disease	Outcome
40–44	M	Yes	IPAA	9	≤ 6 months	Foveolar gastric adenoma	3 (8)	Proximal	80 mm	Adenocarcinoma	Gastrectomy	pT4a, pN3b (20/42), L1, V1, G2, R0	No	Deceased 5 months after initial diagnosis
40–44	M	Yes	IPAA	3	6–12 months	Fundic gland polyps without dysplasia	1 (3)	Proximal	80 mm	Foveolar adenoma with HGD	Gastrectomy	pT1a, pN0 (0/28), L0, V0, G1, R0	No	Deceased 12 months after initial diagnosis due to subsequent cholangiocarcinoma
40–44	F	Yes	IPAA	1	≤ 6 months	Foveolar gastric adenoma	3 (8)	Proximal	20 mm	Adenocarcinoma	NA	NA	Yes (liver)	Deceased 9 months after initial diagnosis
75–79	M	Yes	IPAA	1	> 36 months	Fundic gland polyps without dysplasia	3 (8)	Proximal	50 mm	Adenocarcinoma	NA	NA	Yes (liver)	Deceased 2 months after initial diagnosis
40–44	F	Yes	IPAA	9	6–12 months	Foveolar gastric adenoma	3 (7)	Proximal	60 mm	Adenocarcinoma	Gastrectomy	pT1a, pN0 (0/28), L0, V0, G1, R0	No	Alive
60–64	M	Yes	IPAA	5	6–12 months	Gastric adenoma	2 (5)	Distal	35 mm	Adenocarcinoma	EMR	pT1a, Nx	No	Alive

*Note:* Index histology refers to histology at initial endoscopic diagnosis; final pathology reflects surgical or endoscopic resection specimens when available. Proximal location refers to fundus or corpus. Spigelman stage recorded at time of last surveillance endoscopy before cancer diagnosis.

Abbreviations: EMR = endoscopic mucosal resection; HGD = high‐grade dysplasia; IPAA = ileal pouch–anal anastomosis; LGD = low‐grade dysplasia.

All patients with proximal gastric carcinoma had a severe FAP phenotype and a history of total proctocolectomy with ileal pouch–anal anastomosis. Median age at diagnosis was 44 years (range 42–76). Proximal tumors presented as large lesions (median diameter 60 mm), and outcomes were poor, with 3 of 5 patients dying during follow‐up. One additional patient died later from cholangiocarcinoma.

In contrast, the single antral carcinoma (pT1a) was detected at an early stage and successfully treated endoscopically. The patient remains alive and disease‐free.

### 3.6 Factors Associated With Gastric Dysplasia

Multivariable logistic regression analyses using patient‐level ever/never outcomes are shown in Table 5.

**TABLE 5:** Multivariable logistic regression analyses of factors associated with gastric dysplasia outcomes (*n* = 299).

Predictor	Fundus/Corpus dysplasia OR (95% CI)		Antral dysplasia OR (95% CI)		Any gastric dysplasia OR (95% CI)	
	OR (95% CI)	*p* value	OR (95% CI)	*p* value	OR (95% CI)	*p* value
Age (per 10‐year increase)	1.03 (0.87–1.22)	0.72	0.98 (0.81–1.19)	0.88	0.92 (0.79–1.08)	0.33
Male sex	1.22 (0.74–2.01)	0.43	2.26 (1.28–3.98)	0.005	1.75 (1.08–2.81)	0.022
Baseline FGP > 200	3.39 (1.40–8.19)	0.007	0.81 (0.30–2.21)	0.68	2.40 (0.98–5.89)	0.056
*Helicobacter pylori* infection	0.53 (0.13–2.13)	0.37	0.71 (0.14–3.66)	0.68	0.74 (0.21–2.59)	0.63
PPI use (baseline)	0.93 (0.50–1.74)	0.82	2.64 (1.36–5.12)	0.004	1.69 (0.92–3.11)	0.09
Ever antral dysplasia	2.64 (1.36–5.12)	0.004	NA	NA	1.98 (1.12–3.52)	0.020

*Note:* Outcomes: ever fundus/corpus dysplasia, ever antral dysplasia, and ever any gastric dysplasia (each: ever vs. never). Age modeled per 10‐year increase. All variables were entered simultaneously into each model. Cross‐compartment dysplasia variables were included only in biologically plausible models.

Abbreviations: CI = confidence interval; FGP = fundic gland polyp; OR = odds ratio; PPI = proton pump inhibitor.

For fundic/corpus dysplasia, a baseline FGP burden > 200 polyps was independently associated with dysplasia, as was the presence of antral dysplasia. Age, sex, *Helicobacter pylori* status, and proton pump inhibitor (PPI) use were not significant predictors.

For antral dysplasia, male sex and PPI use were independently associated with dysplasia, whereas age, *H. pylori* status, and fundic gland polyp burden were not. The presence of fundic/corpus dysplasia was independently associated with antral dysplasia. No monotonic association between Spigelman score or stage and gastric dysplasia was observed.

In a global model assessing any gastric dysplasia, male sex was independently associated with dysplasia, whereas a baseline FGP burden > 200 polyps showed a borderline association.

### 3.7 Endoscopic Management and Procedure‐Related Outcomes

Endoscopic therapy was performed in 233 patients (77.9%), comprising a total of 860 resection procedures. Cold snare polypectomy was the predominant technique (*n* = 790; 91.9%), particularly for small FGPs and low‐grade lesions. Larger lesions were treated using endoscopic mucosal resection (EMR; *n* = 63; 7.3%), most often in a piecemeal fashion. Endoscopic submucosal dissection (ESD) was only performed in a minority of cases (*n* = 7; 0.8%).

Regarding resection completeness, en bloc resection was achieved in 549/638 fundus/corpus resections (86.1%) and 207/222 antral resections (93.2%). Piecemeal resection occurred in 87/638 fundus/corpus resections (13.6%) and 11/222 antral resections (5.0%).

Procedure‐related adverse events were uncommon. Minor intraprocedural bleeding occurred in 13 resections (1.5%) and was managed endoscopically without clinical sequelae. Major bleeding occurred in 3 resections (0.3%) and required short‐term intensive care unit monitoring; all cases were successfully controlled endoscopically. No perforations or procedure‐related deaths were observed.

## 4 Discussion

In this longitudinal cohort of FAP patients with structured surveillance, we demonstrate that gastric disease is dynamic, compartment‐specific, and clinically relevant. Progressive fundic and corpus gland polyp burden is common, precedes dysplasia, and is associated with neoplastic progression, whereas antral neoplasia follows a more indolent course with limited malignant potential.

Current international guidelines endorse regular upper gastrointestinal surveillance in FAP, historically focused on duodenal risk stratification, while gastric surveillance is typically less granular and largely lesion‐based [14, 15, 18]. Our data indicate that such an approach may underestimate the importance of the proximal stomach. Fundic gland polyposis was already present in more than 80% of patients at baseline, consistent with contemporary Western cohorts [9]. Importantly, our longitudinal analyses show that polyp burden increases substantially over time, with more than half of patients with 5‐year follow‐up developing extensive polyposis exceeding 100 FGPs. This progressive phenotype is not captured by cross‐sectional assessments [6].

The close association between high polyp burden and fundic/corpus dysplasia observed in our cohort provides a biologically and clinically plausible risk marker. A threshold of more than 200 polyps emerged as a robust predictor of dysplasia, independent of age, sex, *Helicobacter pylori* status, or proton pump inhibitor use. Sensitivity analyses using alternative thresholds (> 100, > 200, > 400 polyps) and an ordinal dose‐response model confirmed that this association is not dependent on any single cut‐point (Supporting Information S1: Table S8). This phenotype‐driven concept is concordant with observations from major Western expert centers that neoplasia in FAP often emerges in the setting of extensive proximal polyposis (“carpeting,” mounds) rather than as isolated lesions [6, 7, 11]. These findings align with Western expert‐center data showing clustering of gastric cancer in patients with high‐risk endoscopic features [9]. From a guideline perspective, FGP burden represents a simple, endoscopically assessable feature that could be readily incorporated into surveillance algorithms.

Although the absolute incidence of proximal gastric carcinoma was low in our cohort, outcomes were poor, with most affected patients presenting with advanced disease and high mortality. A recent nationwide Dutch cohort study of 1230 FAP patients demonstrated a 12‐fold increased gastric cancer risk compared with the general population and reported that since 2020, gastric cancer has surpassed colorectal cancer as the most frequently diagnosed FAP‐associated malignancy, with 43% of gastric cancers presenting at stage IV despite surveillance [19]. This pattern directly underscores the clinical importance of phenotypic risk stratification: identifying patients with high FGP burden and progressive dysplasia is clinically meaningful precisely because early neoplasia is difficult to detect within extensive FGP carpeting.

In contrast, antral neoplasia displayed a distinct biological behavior in our cohort. This is consistent with prior work suggesting that antral adenomas in FAP are often subtle, less frequent, and typically follow a benign course, with malignant transformation being rare and endoscopic cure usually feasible when cancer is detected [20]. Antral adenomas were less frequent, progressed slowly, and rarely transformed into carcinoma. The single antral cancer detected in our cohort was identified at an early stage and successfully treated endoscopically, consistent with the outcomes reported in a prior series [21]. Risk factors for antral dysplasia differed from those of the fundus, with male sex and PPI use emerging as independent predictors (Table 5), whereas polyp burden was not associated. The PPI–antral dysplasia association should be interpreted with caution, as confounding by indication cannot be excluded in this retrospective cohort, and baseline antral dysplasia was present in 12.3% of PPI users versus 9.0% of non‐users (*p* = 0.625); this finding is hypothesis‐generating only. These findings support compartment‐specific risk stratification rather than pooled gastric endpoints. An important observation with implications for surveillance strategy is the bidirectional association between fundic/corpus and antral dysplasia. Although biologically distinct, dysplasia in one gastric compartment was associated with an increased likelihood of dysplasia in the other, suggesting a shared susceptibility in a subset of patients. Nevertheless, the markedly different malignant potential reinforces the need for compartment‐specific risk stratification rather than pooled gastric endpoints, a distinction that is not consistently emphasized in current guidelines.

Endoscopic management outcomes further underscore these differences. Endoscopic resection was effective for antral lesions, with no observed recurrences following complete resection, supporting current recommendations favoring endoscopic therapy in this setting. In contrast, recurrence and metachronous dysplasia were frequent after resection of fundic lesions, particularly when the lesions were large and piecemeal resected. In line with this, recent data from Amsterdam highlight that proximal dysplastic lesions often occur on a background of carpeting fundic gland polyposis and can be challenging to detect and manage endoscopically [7]. From a guideline standpoint, this raises the question of whether repeated local resections meaningfully alter cancer risk in patients with extensive proximal disease.

A key strength is the contemporary cohort (2019 onwards), reflecting current awareness of gastric cancer risk and systematic phenotype documentation. Our center is the largest FAP center in Germany with nationwide referrals; while referral center bias cannot be fully excluded, 56.6% of patients had an FGP burden ≤ 50 polyps or no FGPs at baseline, suggesting the full phenotypic spectrum is represented.

Limitations include the observational design, the inherent subjectivity of endoscopic polyp burden estimation (though sensitivity analyses across multiple thresholds and an ordinal trend model confirmed threshold‐independent associations; Supporting Information S1: Table S8), the potential for surveillance intensity to influence dysplasia detection (formally addressed by adjustment for total endoscopy number and mean surveillance interval, with associations remaining significant; Supporting Information S1: Table S9), the absence of systematic APC variant characterization precluding genotype–phenotype analyses, the small number of gastric adenocarcinoma cases (*n* = 6) precluding cancer‐specific risk modeling (carcinoma data are descriptive and hypothesis‐generating), the absence of external central pathology review (inherent to retrospective studies; all specimens evaluated by a dedicated GI pathology team), and potential referral center bias (findings most directly applicable to specialized hereditary tumor centers). Administrative censoring rather than selective dropout explains the reduction in patient numbers over time; a formal baseline comparison confirmed no significant phenotypic differences between patients with complete and incomplete follow‐up (Supporting Information S1: Table S10).Taken together, our data argue for a refinement of current surveillance recommendations in FAP. Rather than relying solely on lesion size or histology, surveillance intensity should incorporate longitudinal phenotype evolution, particularly progressive FGP burden. Patients with extensive or rapidly increasing proximal polyposis may benefit from shorter surveillance intervals, systematic endoscopic resection, or referral to specialized centers, whereas patients with isolated antral adenomas can be managed according to established endoscopic principles.

In conclusion, gastric manifestations in FAP are not uniform. The progressive FGP burden is associated with a high‐risk phenotype for dysplasia and proximal gastric carcinoma, whereas antral neoplasia follows a more indolent and endoscopically manageable course. These findings support phenotype‐driven, compartment‐specific surveillance strategies and may inform risk stratification at specialized centers and contribute to the evidence base for future guideline refinement.

## Conflicts of Interest

R.H.: Research: Deutsche Krebshilfe, Wilhelm‐Sander‐Stiftung; Speaker: M.S.D., Fujifilm Europe; Other: Endoscopic equipment on loan by Fujifilm. J.N.: Research: Deutsche Krebshilfe, Wilhelm‐Sander‐Stiftung; Other: Endoscopic equipment on loan by Fujifilm. The other authors declare no known conflicts of interest.

## Supporting information


Supporting Information S1

## Data Availability

The data that support the findings of this study are available from the corresponding author upon reasonable request.
