# Supplementary material for: Longitudinal Surveillance of Gastric Polyposis in Familial Adenomatous Polyposis: Incidence, Progression, and Endoscopic Outcomes
Source: United European Gastroenterol J. 2026 Apr 13;14(3):e70216. doi: 10.1002/ueg2.70216 (PMC13072047; doi:10.1002/ueg2.70216)
Supplement: Supplementary file 1 — Supporting Information S1 [file UEG2-14-e70216-s001.docx]

Table S1. Annual point prevalence of fundus/corpus dysplasia during follow-up

Cells show n (% of patients undergoing surveillance endoscopy in the respective follow-up year). Annual point prevalence reflects the proportion of patients with histologically confirmed low-grade or high-grade dysplasia in the fundus or corpus at that surveillance examination. Patients could contribute to more than one follow-up year. Annual prevalence differs from cumulative incidence. LGD = low-grade dysplasia; HGD = high-grade dysplasia. Patients with concurrent LGD and HGD within the same surveillance examination were counted once in the “Any dysplasia” category.

| **Year** | **n** | **LGD, n (%)** | **HGD, n (%)** | **Any dysplasia, n (%)** |
| --- | --- | --- | --- | --- |
| Year 0 | 299 | 32 (10.7%) | 0 (0.0%) | 32 (10.7%) |
| Year 1 | 179 | 25 (14.0%) | 1 (0.6%) | 25 (14.0%) |
| Year 2 | 198 | 34 (17.2%) | 2 (1.0%) | 35 (17.7%) |
| Year 3 | 153 | 31 (20.3%) | 2 (1.3%) | 32 (20.9%) |
| Year 4 | 110 | 28 (25.5%) | 3 (2.7%) | 30 (27.3%) |
| Year 5 | 90 | 35 (38.9%) | 2 (2.2%) | 36 (40.0%) |

Table S2. Fundus/corpus dysplasia incidence in patients with complete 5-year follow-up (n = 90)

Cells show n (% of patients with complete 5-year follow-up, n=90). 'New' indicates first occurrence since the previous surveillance interval. 'Any dysplasia' includes low-grade or high-grade dysplasia detected in the fundus or corpus. LGD = low-grade dysplasia; HGD = high-grade dysplasia.

| **Year** | **n** | **New LGD** | **Cumulative LGD** | **New HGD** | **Cumulative HGD** | **New Any** | **Cumulative any dysplasia** |
| --- | --- | --- | --- | --- | --- | --- | --- |
| Year 0 | 90 | 8 (8.9%) | 8 (8.9%) | 0 (0.0%) | 0 (0.0%) | 8 (8.9%) | 8 (8.9%) |
| Year 1 | 90 | 2 (2.2%) | 10 (11.1%) | 0 (0.0%) | 0 (0.0%) | 2 (2.2%) | 10 (11.1%) |
| Year 2 | 90 | 4 (4.4%) | 14 (15.6%) | 0 (0.0%) | 0 (0.0%) | 4 (4.4%) | 14 (15.6%) |
| Year 3 | 90 | 5 (5.6%) | 19 (21.1%) | 0 (0.0%) | 0 (0.0%) | 5 (5.6%) | 19 (21.1%) |
| Year 4 | 90 | 6 (6.7%) | 25 (27.8%) | 0 (0.0%) | 0 (0.0%) | 6 (6.7%) | 25 (27.8%) |
| Year 5 | 90 | 17 (18.9%) | 42 (46.7%) | 2 (2.2%) | 2 (2.2%) | 19 (21.1%) | 44 (48.9%) |

Table S3. Annual point prevalence of antral dysplasia during longitudinal surveillance. Cells show n (% of patients examined in the respective year). 'Any dysplasia' includes low-grade or high-grade dysplasia detected in the antrum. Annual prevalence is estimated using a ±6-month window around each integer year of follow-up. LGD = low-grade dysplasia; HGD = high-grade dysplasia.

| **Year** | **n** | **LGD, n (%)** | **HGD, n (%)** | **Any dysplasia, n (%)** |
| --- | --- | --- | --- | --- |
| Year 0 | 299 | 27 (9.0%) | 0 (0.0%) | 27 (9.0%) |
| Year 1 | 179 | 20 (11.2%) | 1 (0.6%) | 21 (11.7%) |
| Year 2 | 198 | 16 (8.1%) | 0 (0.0%) | 16 (8.1%) |
| Year 3 | 153 | 12 (7.8%) | 2 (1.3%) | 13 (8.5%) |
| Year 4 | 110 | 8 (7.3%) | 0 (0.0%) | 8 (7.3%) |
| Year 5 | 90 | 12 (13.3%) | 0 (0.0%) | 12 (13.3%) |

Table S4. Cumulative incidence of antral dysplasia in patients with complete 5-year follow-up (n = 90)

Cells show n (% of patients with complete 5-year follow-up, n=90). Cumulative incidence reflects first detection of dysplasia during follow-up. LGD = low-grade dysplasia; HGD = high-grade dysplasia.

| **Year** | **n** | **New LGD** | **Cumulative LGD** | **New HGD** | **Cumulative HGD** | **New any dysplasia** | **Cumulative any dysplasia** |
| --- | --- | --- | --- | --- | --- | --- | --- |
| Year 0 | 90 | 7 (7.8%) | 7 (7.8%) | 0 (0.0%) | 0 (0.0%) | 7 (7.8%) | 7 (7.8%) |
| Year 1 | 90 | 2 (2.2%) | 9 (10.0%) | 0 (0.0%) | 0 (0.0%) | 2 (2.2%) | 9 (10.0%) |
| Year 2 | 90 | 1 (1.1%) | 10 (11.1%) | 0 (0.0%) | 0 (0.0%) | 1 (1.1%) | 10 (11.1%) |
| Year 3 | 90 | 3 (3.3%) | 13 (14.4%) | 2 (2.2%) | 2 (2.2%) | 4 (4.4%) | 14 (15.6%) |
| Year 4 | 90 | 3 (3.3%) | 16 (17.8%) | 0 (0.0%) | 2 (2.2%) | 3 (3.3%) | 17 (18.9%) |
| Year 5 | 90 | 5 (5.6%) | 21 (23.3%) | 0 (0.0%) | 2 (2.2%) | 5 (5.6%) | 22 (24.4%) |

Table S5. Annual point prevalence of any gastric dysplasia across gastric compartments (fundus/corpus or antrum)

Cells show n (% of patients examined in the respective year). 'Any dysplasia' indicates the presence of low-grade or high-grade dysplasia in either the fundus/corpus or the antrum; patients with dysplasia in both compartments were counted once. Annual prevalence is estimated using a ±6-month window around each integer year of follow-up. LGD = low-grade dysplasia; HGD = high-grade dysplasia.

| **Year** | **n** | **LGD, n (%)** | **HGD, n (%)** | **Any dysplasia, n (%)** |
| --- | --- | --- | --- | --- |
| Year 0 | 299 | 52 (17.4%) | 0 (0.0%) | 52 (17.4%) |
| Year 1 | 179 | 39 (21.8%) | 2 (1.1%) | 40 (22.3%) |
| Year 2 | 198 | 48 (24.2%) | 2 (1.0%) | 49 (24.7%) |
| Year 3 | 153 | 37 (24.2%) | 4 (2.6%) | 39 (25.5%) |
| Year 4 | 110 | 32 (29.1%) | 3 (2.7%) | 34 (30.9%) |
| Year 5 | 90 | 43 (47.8%) | 2 (2.2%) | 43 (47.8%) |

Table S6. Cumulative incidence of any gastric dysplasia in patients with complete 5-year follow-up (n = 90)

Cells show n (% of patients with complete 5-year follow-up, n=90). Cumulative incidence reflects first detection of gastric dysplasia (in either compartment) during follow-up. Patients were counted once at the time of first diagnosis. 'Any gastric dysplasia' includes low-grade or high-grade dysplasia detected in either the fundus/corpus or the antrum. Baseline values refer only to the subgroup of patients with complete 5-year follow-up and therefore differ from baseline prevalence in the overall cohort.

| **Year** | **New cases** | **Cumulative cases** |
| --- | --- | --- |
| 0 | 14 (15.6%) | 14 (15.6%) |
| 1 | 3 (3.3%) | 17 (18.9%) |
| 2 | 3 (3.3%) | 20 (22.2%) |
| 3 | 6 (6.7%) | 26 (28.9%) |
| 4 | 6 (6.7%) | 32 (35.6%) |
| 5 | 19 (21.1%) | 51 (56.7%) |

Table S7. Cumulative incidence of any gastric dysplasia during longitudinal surveillance (overall cohort, n=299).

Cells show n (% of the overall cohort). Cumulative incidence reflects first detection of gastric dysplasia during follow-up. 'Any gastric dysplasia' includes low-grade or high-grade dysplasia detected in either the fundus/corpus or the antrum; patients were counted once at the time of first diagnosis.

| **Year** | **New cases** | **Cumulative cases** |
| --- | --- | --- |
| 0 | 52 (17.4%) | 52 (17.4%) |
| 1 | 20 (6.7%) | 72 (24.1%) |
| 2 | 25 (8.4%) | 97 (32.4%) |
| 3 | 19 (6.4%) | 116 (38.8%) |
| 4 | 13 (4.3%) | 129 (43.1%) |
| 5 | 19 (6.4%) | 148 (49.5%) |

Table S8. Sensitivity analysis: Association between fundic gland polyp (FGP) burden and gastric dysplasia using alternative threshold definitions and ordinal trend analysis. All models adjusted for age (per 10-year increase), sex, proton pump inhibitor use, and Helicobacter pylori status. Analysis restricted to patients with ≥2 surveillance endoscopies (n=267).

| **FGP Predictor** | **OR** | **95% CI** | **p-value** | **n** |
| --- | --- | --- | --- | --- |
| **Fundus/Corpus Dysplasia** | | | | |
| >100 polyps (dichotomous) | 3.36 | 1.89–5.95 | **<0.001** | 267 |
| ***>200 polyps (dichotomous, primary analysis)**** | **3.75** | 1.45–9.69 | **0.006** | 267 |
| >400 polyps (dichotomous) | 3.98 | 0.77–20.62 | 0.099 | 267 |
| Ordinal category (0–5 scale, trend) | 1.95 | 1.54–2.46 | **<0.001** | 267 |
| **Antral Dysplasia** | | | | |
| >100 polyps (dichotomous) | 1.34 | 0.71–2.53 | 0.360 | 267 |
| ***>200 polyps (dichotomous, primary analysis)**** | **0.93** | 0.34–2.52 | 0.887 | 267 |
| >400 polyps (dichotomous) | 0.25 | 0.03–2.25 | 0.218 | 267 |
| Ordinal category (0–5 scale, trend) | 1.04 | 0.83–1.30 | 0.738 | 267 |

** Primary analysis as reported in Table 4 of the main manuscript. OR, odds ratio; CI, confidence interval; FGP, fundic gland polyp. Ordinal FGP category: 0 = no FGPs, 1 = 1–50, 2 = 51–100, 3 = 101–200, 4 = 201–400, 5 = >400 polyps.*

Table S9. Sensitivity analysis: Association between fundic gland polyp (FGP) burden and gastric dysplasia after adjustment for surveillance intensity. Models are adjusted for age (per 10-year increase), sex, proton pump inhibitor use, and Helicobacter pylori status, with or without additional adjustment for number of endoscopies per patient or mean surveillance interval. Analysis restricted to patients with ≥2 surveillance endoscopies (n=267). OR, odds ratio; CI, confidence interval.

| **Model** | **OR** | **95% CI** | **p-value** | **n** |
| --- | --- | --- | --- | --- |
| **Fundus/Corpus Dysplasia** | | | | |
| ***FGP >200 polyps (dichotomous)*** | | | | |
| **Without adjustment for surveillance intensity** | **3.75** | 1.45–9.69 | **0.006** | 267 |
| Adjusted for number of endoscopies per patient | 2.81 | 1.01–7.86 | **0.049** | 267 |
| Adjusted for mean surveillance interval (years) | 3.69 | 1.42–9.60 | **0.007** | 267 |
| ***FGP ordinal category (0–5 scale, trend)*** | | | | |
| **Without adjustment for surveillance intensity** | **1.95** | 1.54–2.46 | **<0.001** | 267 |
| Adjusted for number of endoscopies per patient | 1.88 | 1.47–2.41 | **<0.001** | 267 |
| Adjusted for mean surveillance interval (years) | 1.97 | 1.55–2.49 | **<0.001** | 267 |
| **Antral Dysplasia** | | | | |
| ***FGP >200 polyps (dichotomous)*** | | | | |
| **Without adjustment for surveillance intensity** | **0.93** | 0.34–2.52 | 0.887 | 267 |
| Adjusted for number of endoscopies per patient | 0.73 | 0.26–2.06 | 0.554 | 267 |
| Adjusted for mean surveillance interval (years) | 0.95 | 0.35–2.56 | 0.913 | 267 |
| ***FGP ordinal category (0–5 scale, trend)*** | | | | |
| **Without adjustment for surveillance intensity** | **1.04** | 0.83–1.30 | 0.738 | 267 |
| Adjusted for number of endoscopies per patient | 0.98 | 0.78–1.24 | 0.892 | 267 |
| Adjusted for mean surveillance interval (years) | 1.04 | 0.83–1.31 | 0.715 | 267 |

*Bold p-values indicate statistical significance (p<0.05). FGP ordinal category: 0 = no FGPs, 1 = 1–50, 2 = 51–100, 3 = 101–200, 4 = 201–400, 5 = >400 polyps.*

Table S10. Baseline characteristics of patients with complete 5-year follow-up (n=90) compared to patients with shorter available follow-up within the study period (n=209). Differences reflect administrative censoring due to later study entry rather than selective clinical dropout. Continuous variables are presented as median (IQR) and compared using the Mann-Whitney U test; categorical variables are presented as n (%) and compared using the chi-square test or Fisher's exact test as appropriate.

|  | **Complete 5-year follow-up (n=90)** | **Shorter follow-up (n=209)** | **p-value** |
| --- | --- | --- | --- |
| Age, years (median, IQR) | 40 (25.5–52.0) | 36 (26.0–50.0) | 0.521 |
| Male sex, n (%) | 39 (43.3%) | 84 (40.2%) | 0.705 |
| FGP count (median, IQR) | 50 (20–150) | 50 (15–150) | 0.970 |
| FGP >200, n (%) | 8 (8.9%) | 18 (8.6%) | 1.000 |
| F/C dysplasia at baseline, n (%) | 8 (8.9%) | 24 (11.5%) | 0.684 |
| Antral dysplasia at baseline, n (%) | 7 (7.8%) | 20 (9.6%) | 0.826 |
| PPI use, n (%) | 23 (25.6%) | 39 (18.7%) | 0.233 |
| Prior colorectal surgery, n (%) | 70 (77.8%) | 133 (63.6%) | 0.023 |
| No. of endoscopies (median, IQR) | 6 (5–8) | 3 (2–4) | <0.001 |

*Bold p-values indicate statistical significance (p<0.05). FGP, fundic gland polyp; IQR, interquartile range; PPI, proton pump inhibitor. The study period spanned January 2019 to December 2023; patients enrolled later in the study period had fewer years of available follow-up by the time of data cut-off, resulting in a smaller number of patients with 5-year data.*
